# Association between Genetic Variants of *CELSR2-PSRC1-SORT1* and Cardiovascular Diseases: A Systematic Review and Meta-Analysis

**DOI:** 10.3390/jcdd10030091

**Published:** 2023-02-21

**Authors:** Rosa Giannina Castillo-Avila, Thelma Beatriz González-Castro, Carlos Alfonso Tovilla-Zárate, José Jaime Martínez-Magaña, María Lilia López-Narváez, Isela Esther Juárez-Rojop, Pedro Iván Arias-Vázquez, Verónica Marusa Borgonio-Cuadra, Nonanzit Pérez-Hernández, José Manuel Rodríguez-Pérez

**Affiliations:** 1 División Académica de Ciencias de la Salud, Universidad Juárez Autónoma de Tabasco, Villahermosa 86100, Mexico; 2 División Académica Multidisciplinaria de Jalpa de Méndez, Universidad Juárez Autónoma de Tabasco, Jalpa de Méndez 86205, Mexico; 3 División Académica Multidisciplinaria de Comalcalco, Universidad Juárez Autónoma de Tabasco, Comalcalco 86658, Mexico; 4 Laboratorio de Genómica de Enfermedades Psiquiátricas y Neurodegenerativas, Instituto Nacional de Medicina Genómica, Ciudad de México 14610, Mexico; 5 Hospital Chiapas Nos Une Dr. Gilberto Gómez Maza, Secretaría de Salud de Chiapas, Tuxtla Gutiérrez 29045, Mexico; 6 Departamento de Medicina Genómica, Instituto Nacional de Rehabilitación Luis Guillermo Ibarra, Ciudad de México 14389, Mexico; 7 Departamento de Biología Molecular, Instituto Nacional de Cardiología Ignacio Chávez, Ciudad de México 14080, Mexico

**Keywords:** *CELSR2*, *PSRC1*, *SORT1*, cardiovascular diseases, meta-analysis

## Abstract

A cluster of three genes *CELSR2*, *PSRC1*, and *SORT1* has been associated with cardiovascular diseases. Thus, the aim of this study was (i) to perform a systematic review and updated meta-analysis of the association of three polymorphisms (rs646776, rs599839, and rs464218) of this cluster with cardiovascular diseases, and (ii) to explore by PheWAS signals of the three SNPs in cardiovascular diseases and to evaluate the effect of rs599839 with tissue expression by in silico tools. Three electronic databases were searched to identify eligible studies. The meta-analysis showed that the rs599839 (allelic OR 1.19, 95% CI 1.13–1.26, dominant OR 1.22, 95% CI 1.06–1.39, recessive OR 1.23, 95% CI 1.15–1.32), rs646776 (allelic OR 1.46, 95% CI 1.17–1.82) polymorphisms showed an increased risk for cardiovascular diseases. PheWas analysis showed associations with coronary artery disease and total cholesterol. Our results suggest a possible involvement of the *CELSR2-PSRC1-SORT1* cluster variants in the risk association of cardiovascular diseases, particularly coronary artery disease.

## 1. Introduction

Nowadays, cardiovascular diseases (CVDs) are defined as the “pathological processes involving the cardiovascular system such as the heart, the blood vessels, or the pericardium” by the Medical Subject Headings criteria (MeSH). The American Heart Association, until 2020, reported that cardiovascular diseases represented the leading cause of mortality worldwide, with approximately 18.6 million deaths and 523 million cases since records began [1]. In this sense, despite technological advances in the diagnosis and treatment of CVDs, they are the principal contributor to disabilities and decreased quality of life in the population [2]. 

Among the cardiovascular diseases of atherosclerotic origin is mainly coronary artery disease [3]. Therefore, it is important to study several risk factors for CVDs, including dyslipidemias, metabolic syndrome and type 2 diabetes mellitus, and genetics [4,5,6].

Since 2007, various genome-wide association studies (GWAS) have identified risk loci and candidate genes involved in the development of CVDs [7]. In this way, the 1p13.3 locus is of interest because it harbors a group of three candidate genes *CELSR2-PSRC1-SORT1* that could participate in mechanisms that involve the alteration of serum lipids and plasmatic cholesterol levels and increase susceptibility for the development of CVD [8,9].

The *CELSR2* (Cadherin EGF LAG Seven-Pass G-Type Receptor 2) is a member of the flamingo subfamily, part of the cadherin superfamily. This gene encodes a cadherin responsible for contact-mediated cell adhesion and ligand-receptor cell interactions [10]. The *PSRC1* (proline/serine-rich coiled-coil 1) gene is partly responsible for microtubule destabilization [11,12]. The *SORT1* (sortilin) is a gene that encodes the sortilin 1 protein, implicated in several lipid-associated functions such as VLDL secretion, atherosclerotic lesions development, LDL-cholesterol metabolism, and PCSK9 secretion [13]. It also participates in other pathophysiological mechanisms, including inflammation, dyslipidemia, vascular calcification, insulin resistance, and the formation of foam cells [14]. Some of these *SORT1*-associated functions lead to an increased risk of cardiovascular diseases. However, the results regarding these three genes and their participation in CVDs are so far contradictory and not well understood [8,15,16]. Likewise, previously reported associations show the possible role of these genetic variants. However, it is necessary to know the behavior of these polymorphisms in other populations and with updated studies.

Therefore, this study aimed to perform a systematic review and updated meta-analysis of the association of *CELSR2-PSRC1-SORT1* (rs646776, rs599839, and rs464218) with cardiovascular diseases. Finally, we explored prior genome-wide association signals of the rs599839 polymorphism with other phenotypes related to cardiovascular diseases and evaluated the effect of rs599839 on gene expression in the liver using in silico tools.

## 2. Materials and Methods

### 2.1. Databases and Literature Sources

The present work followed the Preferred Reporting Items for Systematic Reviews and Meta-Analyses (PRISMA) guidelines [17] (Supplementary Material S1). This study was also registered and authorized by PROSPERO (International prospective register of systematic reviews), endorsed by the University of York with protocol ID: CRD42021284700. 

A systematic literature search was performed up to December 2021 to identify relevant articles published on three widely used electronic literature databases (PubMed, Web of Science, and Scopus). First, the following keywords were combined to obtain the eligible articles: (i) “rs646776”, “rs599839”, or “rs464218” and (ii) “coronary artery disease”, “cardiovascular disease”, “coronary heart disease”, “cerebrovascular disease”, “myocardial infarction”, “peripheral arterial”, “atherosclerosis”, or “ischemic stroke”. To perform a more detailed search, we also included some cardiovascular risk factors: (i) “rs646776”, “rs599839”, or “rs464218” and (ii) “hypertension”, “lipids”, “metabolic syndrome”, “glucose”, “insulin”, “type 2 diabetes mellitus”, “abdominal obesity”, or “dyslipidemia” in combination with the gene polymorphisms previously mentioned (Supplementary Material S2). 

### 2.2. Eligibility Criteria

Only studies meeting the following inclusive selection criteria were eligible: (1) studies that evaluated human subjects; (2) case-control study designs; (3) studies investigating the association between the genetic variants of *CELSR2-PSRC1-SORT1* (rs646776, rs599839, and rs464218) and cardiovascular disease susceptibility; (4) studies in which genotypes in cases and controls were available or (5) had sufficient data to calculate an odds ratio (OR) with a 95% confidence interval (95% CI); and (6) peer-reviewed studies written in the English language.

The exclusion criteria were meta-analyses, reviews, case reports, abstracts, editorial articles, or duplicate publications with overlapping data. For meta-analyses only, we excluded studies in which the control group had a significant Hardy–Weinberg equilibrium (HWE) *p*-value < 0.05.

Fourteen studies were eligible for systematic review and twelve for meta-analysis following the abovementioned search. 

### 2.3. Assessment of Study Quality 

The quality of the articles included in our study was evaluated using the NOS (Newcastle-Ottawa quality assessment) scale. The evaluation was based on three aspects: the selection (total score of 4), the comparability between groups (total score of 2), and exposure factors (total score of 3), with a total score of 9 points. Studies with a score ≥6 were considered high quality for inclusion in this analysis.

### 2.4. Data Extraction

Two authors (RGCA and TBGC) searched, applied the eligibility criteria and quality assessment, and extracted data from eligible studies. Two other investigators (JMRP and NPH) resolved discrepancies by consensus. 

The information obtained was:

1. last name of the leading author;
2. year of publication;
3. self-reported ancestry and country of study population;
4. number of cases and controls;
5. diagnostic criteria of cases and controls;
6. allele and genotype frequencies.

### 2.5. Methods for Quantitative Synthesis and Statistical Analysis

We calculated the odds ratio and 95% CI to estimate the association between genetic variants of *CELSR2-PSRC1-SORT1* (rs646776, rs599839, and rs464218) and the risk of cardiovascular diseases. Statistical significance was established at a Z-test *p*-value < 0.05. We used the main models of inheritance reported in statistical analyses of genetic polymorphisms in epidemiological studies [18]:

1. Allelic
Allele 1 (Reference)
Allele 2;
2. Codominant
11 (Reference)
12
22;
3. Dominant
11 (Reference)
12 + 22;
4. Recessive
11 + 12 (References)
22.

We evaluated statistical heterogeneity between studies by a chi-square-based Cochran Q test. A Higgins I-squared statistic *p*-value < 0.1 or I^2^ > 50% indicated heterogeneity. We used the random effects model unless otherwise stated, in which case we used the fixed effects model. For a better understanding of CVDs, we performed sub-analyses based on:

- overall, coronary artery disease, myocardial infarction, acute coronary artery syndrome, ischemic stroke, and peripheral arterial disease;
- studies including only coronary artery disease;
- studies including only healthy controls;
- Asian populations.

We replicated the analyses, excluding one study at a time as a sensitivity analysis for the stability of the pooled results. We visualized publication bias by funnel plots, and the quantitative method of Begg/Egger linear regression (*p*-value < 0.05 suggests bias) identified any bias. Chi-squared statistics evaluated Hardy–Weinberg equilibrium (HWE) in each study. All data were analyzed using Comprehensive Meta-Analysis Software version 2. In addition, we duplicated the searches in the different databases: (a) manually by the assigned researchers (RGCA, TBGC, JMRP, and NPH), and (b) by using the COVIDENCE software (https://www.covidence.org/) (accessed on 29 December 2021) to verify searches and articles found. The *p*-value adjusted by the Bonferroni method was considered, and significance was set at *p*-value < 0.004 (*p*-value correction = 0.05/12), and the results were expressed in scientific notation.

### 2.6. Prior Genome-Wide Association Signals with Phenotypes Related to Cardiovascular Diseases

We, additionally, explored previous genome-wide association signals of rs599839 with other cardiovascular disease-related phenotypes considering that this genetic variant has shown the highest number of associations in previous sub-analyses. Therefore, we performed a query of prior associations of rs599839 with related phenotypes in the web tool PheWAS of the GWAS Atlas portal in order to identify any association of this variant with other CAD risk factors [19]. GWAS Atlas is a curated web repository of prior genome-wide associated variants to different phenotypes. We filtered the association based on p-values and phenotypes previously associated with cardiovascular diseases.

### 2.7. Effect of Genetic Variants in the Expression of CELSR2-PSRC1-SORT1 Cluster Transcripts in the Liver

Liver expression of the *CELSR2-PSRC1-SORT1* genes is essential for lipid metabolism. Therefore, we explored the GTeX portal for associations of the rs599839 with the transcripts of the *CELSR2-PSRC1-SORT1* cluster.

## 3. Results

### 3.1. Systematic Review

#### Study Characteristics

**Figure 1:**
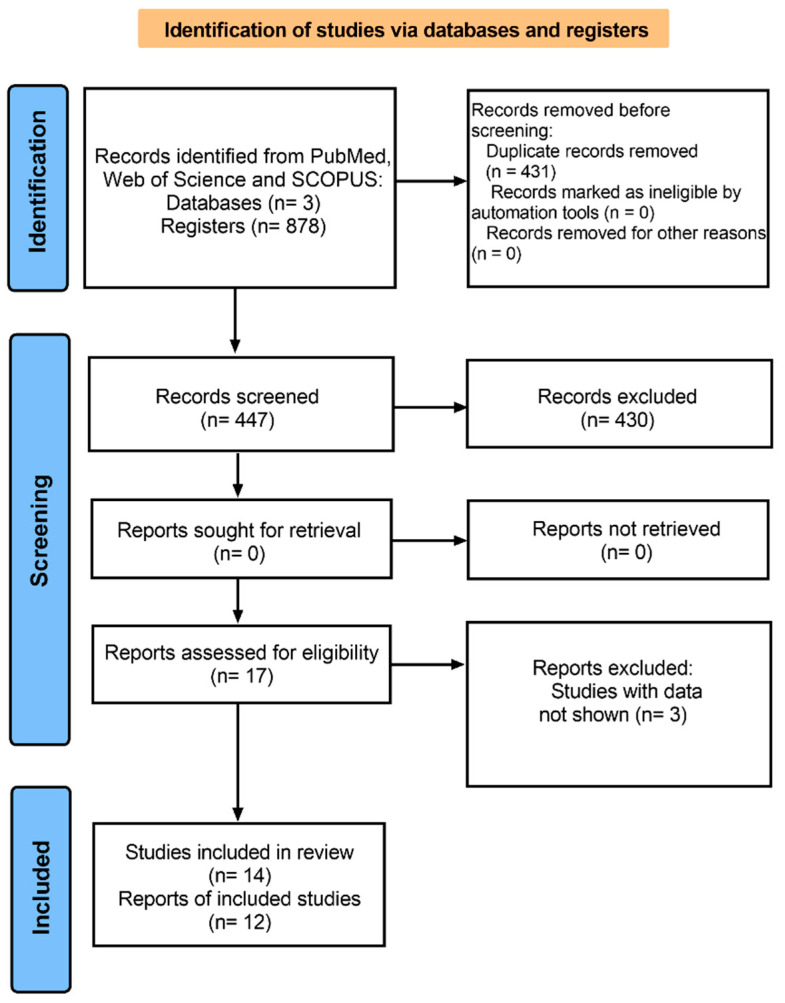
Flow chart showing study inclusion and exclusion details.

A total of 878 articles (306 from PubMed, 305 from Web of Science, and 267 from Scopus) were identified. The flow chart for the study selection is shown in Figure 1. The detailed characteristics of the studies included in the systematic review are shown in Table 1. Of the 14 included studies, based on the country, we observed the following distribution: 4 in China [15,20,21,22], 4 in Japan [23,24,25,26], 1 in New Zealand [27], 1 in Germany [28], 1 in Arabia [29], 1 in Stockholm [30], 1 in the USA [31], and 1 in Pakistan [32]. We found a differential distribution of sample sizes and ages for each SNP. The analysis of the rs599839 polymorphism included 21,553 cases and 29,985 controls. The observed mean age was 61.37 for cases and 59.36 years for controls. The analysis of the rs646776 polymorphism included 2356 cases and 2505 controls, with a mean age of 55.24 years for cases and 53.42 for controls. Finally, for the rs464218 polymorphism, 1315 cases and 691 controls were included. 

All the included studies were of quality on the NOS scale (score > 6).

### 3.2. Meta-Analysis

The quantitative analysis excluded two studies, by Ellis, K.L., 2011 and by Rizk, N. M., 2015, because the control group had a *p*-value < 0.05 in HWE. Subsequently, twelve studies were taken into consideration for further analysis. 

#### 3.2.1. rs599839 Polymorphism and Susceptibility to Cardiovascular Diseases

From the twelve studies included in this meta-analysis, eleven reported genotype data for the rs599839. We performed various sub-analyses for the rs599839 polymorphism as a risk of CVDs. The first analysis was for the overall cardiovascular diagnoses after excluding heterogeneity; we found statistically significant associations in the following genetic models: allelic (OR: 1.19, 95% CI: 1.13–1.26, *p*-value 1 × 10^−4^, Q test *p*-value: 0.175, I^2^: 30.38), dominant (OR: 1.22, 95% CI: 1.06–1.39, *p*-value 3 × 10^−3^, Q test *p*-value: 0.141, I^2^: 34.63), and recessive (OR: 1.23, 95% CI: 1.15–1.32), *p*-value 1 × 10^−4^, Q test *p*-value: 0.423, I^2^: 1.30) (Table 2).

In a sub-analysis, we took into consideration the studies that specifically evaluated coronary artery disease and its relation to the rs599839 polymorphism. The findings without heterogeneity revealed a statistical association with CAD in all the genetic models: allelic (OR: 1.17, 1.10–1.24, *p*-value 1 × 10^−4^, Q test *p*-value: 0.265, I^2^: 21.54), codominant (OR: 1.39, 1.18–1.65, *p*-value 1 × 10^−4^, Q test *p*-value: 0.325, I^2^: 13.66), dominant (OR: 1.24, 1.08–1.42, *p*-value 2 × 10^−3^, Q test *p*-value: 0.153, I^2^: 36.11), and recessive (OR: 1.20, 1.11–1.29, *p*-value 1 × 10^−4^, Q test *p*-value: 0.645, I^2^: 0.000) (Table 2).

Lastly, in an additional sub-analysis, we explored the association of rs599839 polymorphism and CVDs in Asian populations. In this sub-analysis without the presence of heterogeneity, we observed a statistically significant relation in the following genetic models: allelic (OR: 1.27, 1.18–1.36, *p*-value 1 × 10^−4^, Q test *p*-value: 0.494, I^2^: 0.000), and recessive (OR: 1.30, 1.21–1.40, *p*-value 1 × 10^−4^, Q test *p*-value: 0.629, I^2^: 0.000) (Table 2).

#### 3.2.2. rs646776 Polymorphism and Susceptibility to Cardiovascular Diseases

For the studies that genotyped the rs646776 polymorphism, we first evaluated the overall cardiovascular diagnoses. However, the findings revealed no statistical association in the allelic genetic model (OR: 1.17, 95% CI: 0.86–1.59, *p*-value 0.290, Q test *p*-value: 0.040, I^2^: 68.81). Then, in another sub-analysis, we evaluated studies performed in the Asian population. In this sense, the outcomes evidenced a statistical association in the following model: allelic (OR: 1.46, 95% CI: 1.17–1.82, *p*-value 1 × 10^−3^, Q test *p*-value: 0.643, I^2^: 0.00) (Table 3).

#### 3.2.3. rs464218 Polymorphism and Susceptibility to Cardiovascular Diseases

In the analysis of the studies that included the rs464218 polymorphism, we only analyzed the overall diagnoses due to the limited available reports. In this analysis, we found a statistically significant association in the recessive genetic model (OR: 2.03, 95% CI: 1.19–3.47, *p*-value 9 × 10^−3^, Q test *p*-value: 8 × 10^−3^, I^2^: 79.47) (Table 4). However, after correcting the *p*-value, it does not remain significant.

### 3.3. Publication Bias and Sensitive Analysis

Funnel plots visualized publication bias; nonetheless, no evidence of publication bias was found in the pooled analyses. On the other hand, a sensitivity analysis was conducted to assess the influence of one study on the pooled ORs value (allele and dominant genetic models) and whether the results can be reverted by eliminating the individual study. The result did not change after the leaving-one-out analysis, indicating the stability of the results of this meta-analysis.

### 3.4. Bioinformatic Analysis

**Figure 2:**
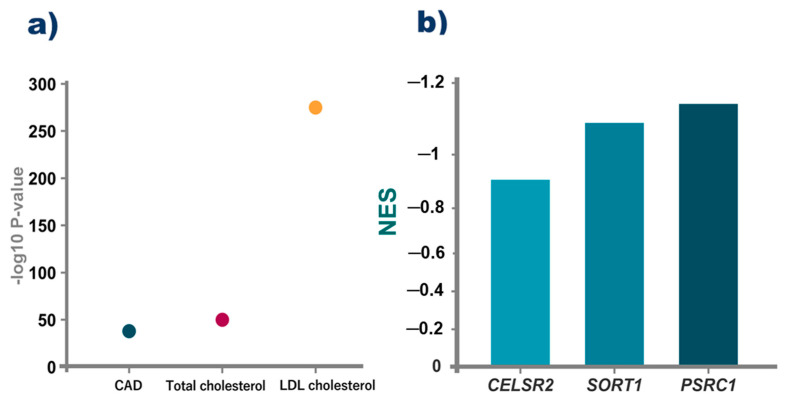
Previous associations of rs599839 with related phenotypes. (**a**) *p*-values for the prior association of rs599839 with coronary artery disease (CAD), total cholesterol, and LDL cholesterol obtained from the PheWAS atlas. (**b**) NES value for the rs599839 on the transcripts liver expression of the genes included on the cluster of *CELSR2-PSRC1-SORT1*. NES is the normalized effect size, which is the slope of the linear regression of the alternative compared to the reference allele.

We performed the bioinformatic analysis only on rs599839, because it showed the highest number of significant associations in the cluster. The results from the PheWAS indicate that this genetic variant had been associated with intermediate phenotypes for CAD, total cholesterol, and LDL levels (Figure 2). Additionally, data from GTeX shows that this variant could change the expression of the three cluster genes in the liver.

## 4. Discussion

Cardiovascular diseases have a complex multifactorial etiology involving various risk factors that contribute to their development, including a genetic component. In this regard, several studies have suggested that genes that together form the *CELSR2-PSRC1-SORT1* gene cluster and are located on chromosome 1 could be involved in the development of cardiovascular diseases through mechanisms related to serum lipid levels and plasma cholesterol levels [8,15].

Therefore, we analyzed three polymorphisms (rs646776, rs599839, and rs464218) in the *CELSR2-PSRC1-SORT1* cluster in 25224 cases and 33181 controls, investigating the association between these genetic variants and susceptibility to develop cardiovascular diseases through a systematic review and updated meta-analysis. 

First, after discarding heterogeneity in the included studies, we observed that the rs599839 could increase the risk of developing any cardiovascular disease (coronary heart disease, myocardial infarction, or acute coronary artery syndrome) by 1.19 to 1.23 times in the allelic, dominant, and recessive models.

Also, when we analyzed the rs599839 polymorphism and coronary artery disease only, we found a strong risk association that ranged from 1.17 to 1.39 times more presenting coronary artery disease in all genetic models evaluated: allelic, codominant, dominant, and recessive. These results suggest that the rs599839 polymorphism may be associated with increased susceptibility to developing coronary artery disease. Previous studies have explored the relationship between the rs599839 polymorphism and coronary artery disease [15,28]. Although we found a strong risk association, Rodríguez-Arellano ME, 2020, reported a protective association with CAD in the Mexican population [16]. These contradictory results could be due to variables that affect the genotype–phenotype association, such as age, ancestry, gene–environment interactions, sex, and sample size. However, we must consider that our CAD sub-analysis included various Asian, American, and European populations, not just one population [15,27,28,31].

Finally, we carried out a specific analysis of Asian populations. When we ruled out heterogeneity, we found that carriers of the allelic and recessive models had 1.27 and 1.30 more times, respectively, the risk of developing cardiovascular diseases. In this regard, Matsuoka, R., 2015 found an association between the rs599839 polymorphism (*PSRC1*, FDR = 0.0118) and myocardial infarction in the Asian population [24]. Our results suggest that the rs599839 polymorphism of the *PSRC1* gene could participate in the development of cardiovascular diseases and mainly in the genetic predisposition to coronary artery disease.

We also analyzed the rs646776 polymorphism of the *CELSR2* gene. In the first analysis, we evaluated the overall diagnoses, and we did not find statistical significance in the allelic model. Then, we performed a second analysis evaluating the Asian population in the included studies, and we observed in the allelic model a higher risk of developing cardiovascular diseases. In this regard, Kathiresan et al., 2009 [33] reported a significant association between rs646776 and early-onset myocardial infarction in 560 controls and 1231 patients from the following countries: Italy, Finland, Boston and Seattle in the United States, Spain, and Sweden. Our results suggest that the rs646776 polymorphism could be associated with an increased risk of cardiovascular diseases in the Asian population.

Moreover, we analyzed the rs464218 polymorphism of the *SORT1* gene. We observed an association that suggests the risk of developing CVDs (overall diagnoses) under the recessive model. There are few studies described in the literature on this polymorphism, and the results are controversial. Therefore, additional studies are required to elucidate the participation of this genetic variant in cardiovascular diseases [15,22]. Although we observed statistical significance, it is important to mention that because of the limited number of studies included in this sub-analysis, we could not rule out heterogeneity. In addition, after correcting *p*-value, the significance does not remain. Therefore, it is possible that the results are biased. Furthermore, this sub-analysis only included the Asian population due to the lack of studies to include other populations. 

On the other hand, our work has important strengths: this is the first meta-analysis involving three genetic variants (rs646776, rs599839, and rs464218) contained in the *CELSR2-PSRC1-SORT1* cluster, and we found an association with different cardiovascular diseases. Second, this work shows a strong risk association between the rs599839 polymorphism and coronary artery disease. Third, in this meta-analysis, we included studies in the main electronic databases and multiple searches to gather as much information as possible. 

We recognize that we have limitations in our study: First, due to the few studies reported in different populations, we were only able to perform a sub-analysis in the Asian population. Second, due to the small sample size for some subgroup analyses, it will be necessary to conduct additional studies with larger samples that include different populations in order to improve the reliability and stability of the meta-analysis. Third, because only studies published in the English language were included, there could be a language bias. Fourth, despite the few studies included in the present meta-analysis, the *p*-value was adjusted by Bonferroni correction. However, this correction could be improved in future studies with more diverse and larger sample sizes, considering other approaches applied to candidate gene studies. Fifth, we did not perform a haplotype analysis due to the few studies included in this meta-analysis. Sixth, we suggest other computational tools and approaches that could be more stringent in calculating statistical significance for future studies. 

## 5. Conclusions

In conclusion, our results suggest an association of the *CELSR2-PSRC1-SORT1* cluster variants with an increased risk of cardiovascular diseases, particularly coronary artery disease. However, our findings warrant replication in larger sample sizes due to several limitations.

## Figures and Tables

**Table 1:** Systematic review of cases and controls about the association between rs599839, rs646776, and rs464218 polymorphisms and cardiovascular diseases.

First Author	Country		Cases					Controls				
		Diagnostic	NOS	N	Male	Female	Age *	Diagnostic	N	Male	Female	Age *
	*rs599839 polymorphism*											
Zhou, Y.J., 2015 [15]	China	CAD	7	561	417	144	62.1 ± 10.5	HC	590	431	159	61.3 ± 9.7
	China	IS	7	527	383	144	62.7 ± 12.3	-	-	-	-	-
Zhou, L., 2011 [20]	China	CHD	8	1269	971	298	60.5 ± 10.4	HC	2745	1844	901	60.6 ± 8.5
Qin, J., 2018 [21]	China	DM+PAD	7	440	223	217	66.7 ± 7.5	NC	442	246	196	60.4 ± 7.9
Ueyama, C., 2015 [23]	Japan	MetS	8	1822	1228	594	64.4 ± 10.3	HC	1096	635	461	63.4 ± 11.7
Matsuoka, R., 2015 [24]	Japan	MI	7	1824	1468	356	64.6 ± 10.3	HC	2329	1029	1300	62.3 ± 11.8
Fujimaki, T., 2015 [25]	Japan	AH	8	3348	2139	1209	66.2 ± 10.2	HC	2112	1162	950	61.9 ± 12.1
Ellis, K.L., 2011 [27]	New Zealand	CD	8	1794	1272	525	66.5 ± 12.3	HC	1649	1093	622	62.8 ± 10.8
Kleber, M.E., 2010 [28]	Germany	CAD	8	2508	1881	627	64 ± 10	HC	681	354	327	58 ± 12
	Germany	CAD, MI	8	1575	1197	378	64 ± 10	-	-	-	-	-
Rizk, N.M., 2015 [29]	Arabia	ACS	8	136	110	26	56.3 ± 10.5	NC	91	68	23	56.86 ± 9.3
Han, W., 2020 [22]	China	CHD	8	227	NA	NA	51.2 ± 6.9	HC	101	NA	NA	49.93 ± 8.0
Gigante, B., 2012 [30]	Stockholm	MI	8	1213	852	361	60 (53–65)	HC	1561	1054	507	61 (54–66)
Bressler, J., 2010 [31]	USA	CHD	7	397	197	200	54.8	HC	3206	1131	2075	53.1
	USA	CHD	7	1362	931	431	55.5	HC	8710	3643	5067	53.9
Abe S, 2015 [26]	Japan	High-LDL-C	8	1174	672	500	63.7 ± 10.7	NC	3296	2146	1150	64.5 ± 11.0
		*Overall*		21,553	14,941	6386	61.37	-	29,985	15,794	14,156	59.36
	*rs646776 polymorphism*											
Ansari, W.M., 2019 [32]	Pakistan	PCAD	8	340	329	11	42 ± 3.8	HC	310	298	12	39 ± 7.8
Qin, J., 2018 [21]	China	DM+PAD	7	440	223	217	66.7 ± 7.5	NC	442	246	196	60.4 ± 7.9
Rizk, N.M., 2015 [29]	Arabia	ACS	8	136	110	26	56.3 ± 10.5	NC	91	68	23	56.8 ± 9.3
Han, W., 2020 [22]	China	CHD	8	227	NA	NA	51.2 ± 6.9	HC	101	NA	NA	49.9 ± 8.0
Gigante, B., 2012 [30]	Stockholm	MI	8	1213	852	361	60 (53–65)	HC	1561	1054	507	61 (54–66)
		*Overall*		2356	1514	615	55.24	-	2505	1666	738	53.42
	*rs464218 polymorphism*											
Zhou, Y.J., 2015 [15]	China	CAD	7	561	417	144	62.1 ± 10.5	HC	590	431	159	61.3 ± 9.7
	China	IS	7	527	383	144	62.7 ± 12.3	-	-	-	-	-
Han, W., 2020 [22]	China	CHD	8	227	NA	NA	51.2 ± 6.9	HC	101	NA	NA	49.9 ± 8.0
		*Overall*		1315	800	288	58.6	-	691	431	159	55.6

* Mean ± standard deviation or median (interquartile range). CAD: coronary artery disease; IS: ischemic stroke; CHD: coronary heart disease; DM: type 2 diabetes mellitus; PAD: peripheral arterial disease; MetS: metabolic syndrome; MI: myocardial infarction; AH: arterial hypertension; CD: coronary disease; ACS: acute coronary artery syndrome; High-LDL-C: hyper-LDL-cholesterolemia. HC: healthy control; NC: Non-cases (<50% stenosis, some comorbidities).

**Table 2:** Association between rs599839 polymorphism and cardiovascular diseases.

Model	Random Effect	Z *p*-Value	Q Test *p*-Value	I^2^	Egger Test *p*-Value
Overall					
G	*Reference*				
A	1.27 (1.11–1.44)	1 × 10^−4^	1 × 10^−4^	80.82	0.100
A *	1.19 (1.13–1.26)	1 × 10^−4^	0.175	30.38	0.108
GG ^c^	*Reference*				
GA ^c^	1.11 (0.92–1.35)	0.28	0.155	31.70	0.100
AA ^c^	1.33 (1.00–1.75)	0.045	0.009	58.90	0.100
GG ^d^	*Reference*				
GA+AA ^d^	1.25 (0.98–1.59)	0.064	0.019	53.15	0.300
GA+AA ^d^*	1.22 (1.06–1.39)	3 × 10^−3^	0.141	34.63	0.446
GG+GA ^r^	*Reference*				
AA ^r^	1.29 (1.12–1.49)	1 × 10^−4^	1 × 10^−4^	76.65	0.103
AA ^r^*	1.23 (1.15–1.32)	1 × 10^−4^	0.423	1.30	0.175
CAD					
G	*Reference*				
A	1.30 (1.11–1.51)	1 × 10^−4^	1 × 10^−4^	81.22	0.100
A *	1.17 (1.10–1.24)	1 × 10^−4^	0.265	21.54	0.310
GG ^c^	*Reference*				
GA ^c^	1.18 (1.00–1.39)	0.039	0.340	11.7	0.100
AA ^c^	1.51 (1.14–2.00)	4 × 10^−3^	0.041	54.2	0.100
AA ^c^*	1.39 (1.18–1.65)	1 × 10^−4^	0.325	13.66	0.614
GG ^d^	*Reference*				
GA+AA ^d^	1.39 (1.08–1.79)	0.010	0.037	52.97	0.104
GA+AA ^d^*	1.24 (1.08–1.42)	2 × 10^−3^	0.153	36.11	0.301
GG+GA ^r^	*Reference*				
AA ^r^	1.32 (1.12–1.56)	1 × 10^−3^	1 × 10^−4^	75.99	0.100
AA ^r^*	1.20 (1.11–1.29)	1 × 10^−4^	0.645	0.000	0.386
Asian population					
G	*Reference*				
A	1.32 (1.12–1.57)	1 × 10^−3^	1 × 10^−4^	80.70	0.290
A *	1.27 (1.18–1.36)	1 × 10^−4^	0.494	0.000	0.236
GG ^c^	*Reference*				
GA ^c^	0.84 (0.60–1.16)	0.288	0.600	0.000	0.340
AA ^c^	1.29 (1.29–1.30)	0.260	0.060	43.48	0.320
AA ^c^*	1.07 (0.77–1.49)	0.676	0.424	1.201	0.653
GG ^d^	*Reference*				
GA+AA ^d^	1.12 (0.81–1.55)	0.466	0.112	37.12	0.323
GG+GA ^r^	*Reference*				
AA ^r^	1.34 (1.14–1.59)	1 × 10^−4^	1 × 10^−4^	77.79	0.324
AA ^r^*	1.30 (1.21–1.40)	1 × 10^−4^	0.629	0.000	0.333

^c^: codominant; ^d^: dominant; ^r^: recessive. * Model without heterogeneity.

**Table 3:** Association between rs646776 polymorphism and cardiovascular diseases.

Model	Random Effect	Z *p*-Value	Q Test *p*-Value	I^2^	Egger Test *p*-Value
Overall					
C	*Reference*				
T	1.17 (0.86–1.59)	0.290	0.040	68.81	0.420
Asian Population					
C	*Reference*				
T	1.46 (1.17–1.82)	1 × 10^−3^	0.643	0.00	0.462

**Table 4:** Association between rs464218 polymorphism and cardiovascular diseases.

Model	Random Effect	Z *p*-Value	Q Test *p*-Value	I^2^	Egger Test *p*-Value
Overall					
G	*Reference*				
A	1.10 (0.85–1.41)	0.448	0.020	74.58	0.101
GG ^c^	*Reference*				
GA ^c^	0.847 (0.37–1.92)	0.692	1 × 10^−4^	94.4	0.100
AA ^c^	1.94 (0.76–4.92)	0.163	1 × 10^−4^	92.2	0.100
GG ^d^	*Reference*				
GA+AA ^d^	1.10 (0.48–2.48)	0.817	1 × 10^−4^	95.27	0.893
GG+GA ^r^	*Reference*				
AA ^r^	2.03 (1.19–3.47)	9 × 10^−3^	8 × 10^−3^	79.47	0.951

^c^: codominant; ^d^: dominant; ^r^: recessive.

## Data Availability

Not applicable.

## Supplementary Materials

The following are available online at https://www.mdpi.com/article/10.3390/jcdd10030091/s1, Supplementary Material S1: PRISMA 2020 Checklist; Supplementary Material S2: Search formulas in the databases included.
